# Mindfulness-Based Stress Reduction Program to Improve Well-Being and Health in Healthcare Professionals

**DOI:** 10.3390/jcm14217655

**Published:** 2025-10-28

**Authors:** Marco Marotta, Niccolo Grassi, Alessandro Pingitore, Alessandra Parlanti, Sergio Berti, Cristina Vassalle

**Affiliations:** 1 Fondazione CNR-Regione Toscana G. Monasterio, 54100 Massa, Italy; 2 Istituto di Fisiologia Clinica, CNR, 56124 Pisa, Italy; 3 Fondazione CNR-Regione Toscana G. Monasterio, Via Moruzzi 1, 56124 Pisa, Italy

**Keywords:** mindfulness, healthcare workers, psycho-emotional status, well-being, distress, PGWBI, PSS, MBI, cardiovascular risk, prevention, care quality, dyslipidemia

## Abstract

**Aim:** To evaluate basal well-being and the effects of a Mindfulness-Based Stress Reduction (MBSR) program in health-care professionals (HCPs), a recognized worker category subjected to elevated stress from job conditions. **Methods:** A quasi-experimental pre–post study was conducted in Italian HCPs. Well-being (assessed by Psychological General Well-Being Index-PGWBI), stress (Perceived Stress Scale-PSS), and burnout (Maslach Burnout Inventory-MBI) were collected at baseline and after an MBSR program. Moreover, levels of C reactive protein, glucose, and lipid profiles were also monitored in a subgroup. **Results:** At baseline, Total-PGWBI score value evidenced no distress, whereas Total-PSS and MBI dimensions (emotional exhaustion—EE, depersonalization and detachment from the job—DP, and lack of personal or professional accomplishment—PA) indicate moderate distress. After MBSR, PGWBI, PSS, and MBI dimensions significantly improved. Moreover, significant benefits on lipid profile were observed after MBSR. **Conclusions:** MBSR may be a promising method to improve well-being and lipid profile in HCPs. Thus, MBSR might represent a new future complementary prevention tool for mental and physical health maintenance in this category of workers.

## 1. Introduction

Healthcare professionals (HCPs) frequently experience high stress levels, depersonalization, emotional exhaustion, and burnout, which not only may affect their health and well-being, but also impacts the quality of structural organization and costs with adverse repercussions on care they provide to patients [1,2]. Thus, in the last few years, the interest for HPC well-being has become a “hot topic”, with a number of studies focused on related challenges and potential solutions.

In this context, mindfulness-based stress reduction (MBSR), originally proposed in patients with chronic diseases to face distress and improving quality of life, has become a psychological strategy utilized worldwide in different clinical fields [3]. Consequently, MBSR practice may represent a useful tool also to face HCP distress. Moreover, MBSR has been associated with variation in the inflammatory, lipid, and glycemic profile in patients with chronic diseases or healthy subjects [4,5,6,7]. In fact, there are different possible physiological mechanisms linking mindfulness practice with improved metabolic parameters or reduced inflammation, which include modulation of autonomic nervous system or stress hormone regulation [4]. Specifically, MBSR can improve the lipid profile reducing stress and the activity of the sympathetic nervous system, whereas it increases parasympathetic nervous system activity and leads to reduction of stress hormones, such as cortisol [4]. Then, these effects can modulate a number of physiological pathways, including fat mobilization and gluconoeogenesis, with benefits on the lipid profile [4]. Interestingly, the increased sympathetic nervous system activity is associated with specific lipids, such as ceramides, sphingomyelins, phosphatidylcholines, and gangliosides, and is independent from insulin resistance [2]. Thus, emerging data indicate that mindfulness practice may significantly affect metabolomic and lipidomic profiles, although the culprit underlying mechanisms and relationship need to be further elucidated [6,7].

In HCPs, the effectiveness of MBSR in mental health and well-being has been consistently documented in a number of reviews and meta-analyses [8,9,10]. However, there is little knowledge/controversial evidence on the efficacy of mindfulness training according to some key determinants (e.g., gender and aging) in general and specifically in HCPs, and lacking data are available on the effects of this practice on metabolic biomarkers in HCPs. In fact, whether women consistently report higher stress levels than men and are generally engaged more in meditation practices (e.g., mindfulness) than men [11,12,13], contrasting data are reported on the existence of a significant relationship of mindfulness with gender [14,15,16,17]. Moreover, the majority of studies on MBSR focus on young people, neglecting older subjects with a greater load of years worked, who could benefit more from this intervention [18,19,20]. More importantly, there is a lack of combined psychological and metabolic assessments, especially in HCPs.

Thus, the present study aimed to evaluate psycho-emotional status and the effects of following an 8-week MBSR program in HCPs, also evaluating possible differences related to gender and aging. Moreover, the putative effects of MBSR intervention on metabolic and inflammatory-related biomarkers, such as blood glucose, C reactive protein (CRP), and lipid profile were also assessed in a subgroup of participants.

## 2. Materials and Methods

### 2.1. Study Sample and Procedures

Fondazione Monasterio (FTGM) is a cardiological center including two hospitals located in two different Tuscany cities (Massa and Pisa, Italy), serving a vast territory of northwestern Tuscany.

**Figure 1:**
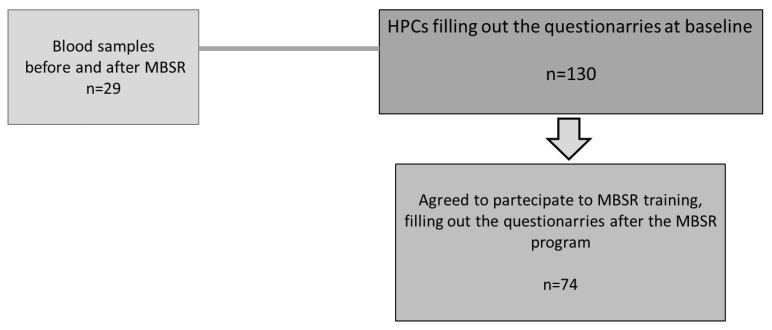
Workflow diagram. HCPs: healthcare professionals; MBSR: mindfulness-based stress reduction.

A total of 130 participants (mean age 42 ± 11, 115 women), including physicians, nurses, and other HCPs from the two FTGM hospitals, voluntarily filled out the questionnaires at baseline. Then, a quasi-experimental pre–post study without randomization was conducted on 74 subjects (mean age 41 ± 12, 22 women), who agreed to follow an MBSR program between 2022 and 2023. Moreover, blood samples were obtained before and after the MBSR training by a subgroup of participants (*n* = 29, 25 females, age 48 ± 8 years). The participant flow diagram is reported in Figure 1.

Inclusion criteria were (a) HCP adults; (b) voluntary participation in the study; (c) questionnaires completed for all data; (d) signed informed consent. Exclusion criteria: acute or chronic inflammatory diseases, immunological, psychiatric, cardiovascular or other systemic diseases.

### 2.2. Ethics Approval and Informed Consent

A written informed consent was obtained prior to participation. The study protocol was approved by the local Ethics Committee (number 19214, 11 February 2021).

### 2.3. Mindfulness-Based Stress Reduction Course

The MBSR technique (proposed by Kabat-Zinn in the 1970s) mixed mindfulness meditation, body awareness, yoga, and deepening behavior, thinking, feeling, and action [21]. MBSR training consists of an 8-week program, with a weekly 2 h group meeting and a recommended 30 min daily meditation practice at home.

One of the authors (M.M.), a psychologist with multi-year experience in practicing and teaching mindfulness meditation, periodically organizes Mindfulness-Oriented Meditation Training courses as a part of a preventive FTGM project to support HCPs’ well-being, as we previously described [18]. The courses are held in a quiet, heated classroom, face-to-face and anonymity guaranteed. Each weekly meeting begins with a brief presentation of the session theme, followed by related, guided experiential exercises, according to the main following activities: (a) teaching on topics related to meditative practice; (b) guided practice; (c) a final stage of sharing experiences. The instructor is available for any problems that emerged for the participants before and after the start of the lessons. Participants are also encouraged to practice at home for 30 min/day.

### 2.4. Questionnaires

HCPs were given the Italian version of validated questionnaires described herein, which are also commonly used in HCP cohorts, as we previously reported [22]. The questionnaires were administered at baseline (T0) to all participants and after (T1) the MBSR training course to 74 subjects. Data on gender, age, physical activity, diet, and occupational category of the participants were also collected.

Psychological General Well-Being Index (PGWBI): A 22-item self-reported questionnaire to measure the level of subjective psychological well-being or discomfort in the previous 4 weeks. The items are rated on a 6-point scale (Likert scale from 0 to 5) for six different dimensions: anxiety (5, 8, 17, 19, 22 items), depressed mood (3, 7, 11), positive well-being (1, 9, 15, 20), self-control (4, 14, 18), general health (2, 10, 13), and vitality (6, 12, 16, 21). A high score is indicative of elevated levels of well-being (maximum value corresponding to 110 points). Scores of questions 1, 4, 6, 7, 9, 10, 14, 16, 19, and 21 were reversed. Global scores < 54 points reflect severe distress, 55–65 moderate distress, and ≥66 indicate a positive PGWBI or “no distress” status.

Perceived Stress Scale (PSS): A self-reported 10-item scale that assesses the perceived intensity of stress over the previous 4 weeks (global perceived stress-PS). Each item is rated on a 5-point Likert scale (0 = never, 4 = very often; maximum value of 40). Scores of questions 4, 5, 7, and 8 were reversed. PS between 0 and 13 considered low, 14 and 26 moderate, and high between 27 and 40.

Maslach Burnout Inventory (MBI): Burnout was measured by using a 7-point Likert scale response format (0 = never, 6 = always), including 22 items that rate the three components of burnout: feelings of overwhelming emotional exhaustion (EE; sum of 9 items: 1, 2, 3, 6, 8, 13, 14, 16, 20), depersonalization and detachment from the job (DP; sum of 5 items: 5, 10, 11, 15, 22), and lack of personal or professional accomplishment (PA; sum of 8 items: 4, 7, 9, 12, 17, 18, 19, 21). Burnout is indicated by high scores for EE and DP, and a low score for PA, as follows: -EE categorized into 0–18 (low), 19–26 (moderate), ≥27 (high); -DP divided into 0–5 (low), 6–9 (moderate), ≥10 (high); -PA classified into 0–33 (high), 34–39 (moderate), ≥40 (low).

Finally, participants were encouraged to write a voluntary free comment reporting reflections on problems at work and their own point of view and thoughts on what creates discomfort, eventually illustrating the possible actions to counteract stressful situations.

International Physical Activity (PA) Questionnaire: Participants’ self-reported PA was assessed at baseline (before the beginning of MBSR training) using the International Physical Activity Questionnaire (IPAQ), a validated instrument utilized to estimate habitual practice of physical activities of people from different countries and socio-cultural contexts [23]. Parameters analyzed included frequency, intensity, and duration of PA; a metabolic equivalent (MET) score is then calculated for each domain to quantify and classify participants’ PA levels. The total weekly MET-minutes (calculated multiplying total minutes/day engaged in activities and the number of days/weeks for MET coefficients that indicate activity intensity) are summed to determine overall activity levels: if <700 MET inactivity, 700–2519 MET active subject, >2519 MET very active subject.

Literature-based adherence score to the Mediterranean diet (MEDI-LITE score): Starting from the studies present in a meta-analysis on the Mediterranean diet and health status published in 2014, the values chosen as a threshold to determine adherence to the Mediterranean diet were first extracted from each individual study [24,25]. Subsequently, the food consumption averages of all the values obtained from the studies were calculated and the values obtained were weighted by the number of subjects included in each study. Then, for each food group considered in the questionnaire, the mean value of all weighted medians was calculated with the addition of 2 standard deviations. The values identified by the 2 standard deviations were chosen as threshold values to define three consumption categories for each food group [24,25]. From this calculation it was possible to obtain daily and/or weekly consumption values for typical foods of the Mediterranean diet (fruit, vegetables, cereals, legumes, and fish) and for non-typical foods (meat and meat products, dairy products). For typical products, 2 points were assigned to the highest consumption category, 1 point to the intermediate category, and 0 points to the lowest category. Conversely, for food products not typical of the Mediterranean Diet (meat and meat products, dairy products) 2 points were assigned to the lowest intake, 1 point to the intermediate intake and 0 points to the highest consumption. For alcohol, the alcohol unit categories (1 alcohol unit = 12 g of alcohol) were used, attributing 2 points to the average category (1–2 alcohol units/day) of consumption, 1 point to the lowest category (1 alcohol unit/day) and 0 points to the highest consumption category (>2 alcohol units/day). Finally, 2 points were awarded for regular use of olive oil, 1 point for frequent use, and 0 points for occasional use. The final score, obtained from the sum of all these values, ranges from 0 (low adherence) to 18 (high adherence).

### 2.5. Blood Samples

Blood samples were collected after an overnight fast and centrifuged at 2500 g for 10 min before analyzing blood glucose, lipid profile (total cholesterol, TotCH; triglycerides, TG; high density lipoproteins, HDL; levels of low density lipoproteins, LDL were calculated with the Friedewald equation), and hsC-reactive protein (hsCRP) with standard automated clinical chemistry laboratory analyzers (Cobas 6000 analyzer, Roche). Laboratory personnel were blinded to the samples.

### 2.6. Statistical Analysis

Data were reported as the mean ± SD, unless stated. Statistical analyses included unpaired or paired Student’s *t*-test (to assess a significant difference between the means of two groups); in particular the unpaired *t*-test was used to assess differences related to aging and gender in the overall population, while the paired *t*-test was used to assess pre–post MBSR differences. Moreover, the χ^2^ test was used to assess the association between two or more variables, when they were categorical.

A post hoc power analysis was conducted (G*Power 3.1 program); a medium effect size of 0.5 was chosen, obtaining a study power (1 − β) of 0.998 with an α value of 0.05.

A significance level of 0.05 was chosen. Statistical analysis was performed using Statview statistical software version 5.0.1 procedures (Abacus Concepts, Berkeley, CA, USA).

## 3. Results

### 3.1. Baseline Characteristics

The studied population consisted of 130 HCPs (age range 21–67 years, 115 women), including physicians (20%), nurses (53%), and other HCPs (psychologists, physiotherapists, technicians, and healthcare assistants; 27%) from the two FTGM hospitals. The mean working experience was 14 ± 10 years. All subjects follow the principles of the Mediterranean diet since all subjects reported a score higher than 8.5 on the MEDI-LITE questionnaire, which suggests a good adherence to the MD [24]. Regarding physical activity, 24% of the overall population was inactive, 55% active, and 21% very active, according to the IPAQ.

### 3.2. Baseline Well-Being, Stress, and Burnout

#### 3.2.1. Psychological General Well-Being Index

***Overall population:*** Values of the 22 items and 6 PGWBI domains and the total score at baseline in the overall population are reported in Table 1 (percent of participants with low, moderate or high levels of total PGWBI score was 19, 18, and 63%, respectively).

**Figure 2:**
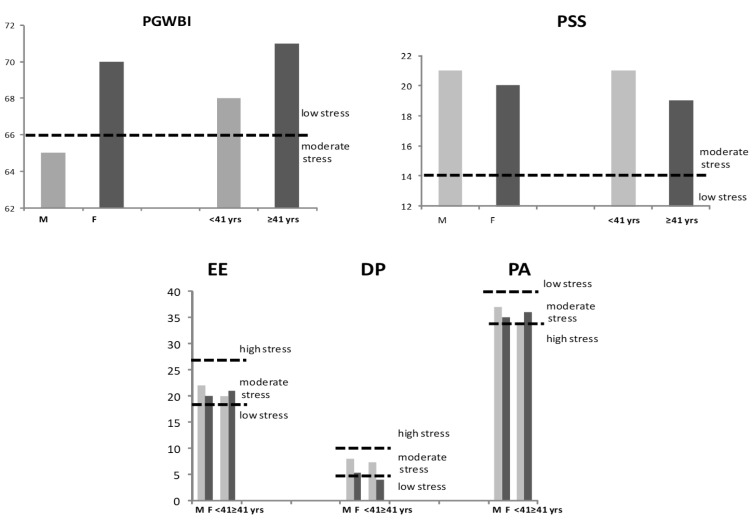
Values of PGWBI, PSS, and MBI (EE, P, and PA) according to gender and age in the HCPs (unpaired *t*-test). PGWBI: Psychological General Well-Being Index; PSS: Perceived Stress Scale; MBI: Maslach Burnout Inventory; EE: emotional exhaustion; DP: depersonalization and detachment from the job; PA: lack of personal or professional accomplishment; HCPs: healthcare professionals; M: males; F: females; yrs: years.

***Sex-related differences:*** The total PGWBI in the two sexes fall in the range of moderate distress for men, and no stress for women (65 ± 21 vs. 70 ± 15, *p* = ns; unpaired *t*-test; Figure 2).

Females compared to males showed significantly higher values for item 11 (“*Have you felt so sad, discouraged, hopeless, or had so many problems that you wondered if anything was worthwhile during the past month?*” in the “depressed mood” dimension; 4.6 ± 0.8 vs. 3.8 ± 1.8, *p* = 0.005) and item 4 (*“I was emotionally stable and sure of myself during the past month”* in the self-control” dimension; 3.1 ± 1.2 vs. 2.5 ± 1.2, *p* = 0.05) (unpaired *t*-test).

***Aging effects:*** When the population was divided according to the 50th percentile (<41 years), the total PGWBI in the two age subgroups fall in the range of no stress (68 ± 15 vs. 71 ± 16, young vs. elderly subjects, *p* = ns) (Figure 2; unpaired *t*-test).

The only significant difference between the items was observed regarding item 8 in the “anxiety” dimension (*“Were you generally tense-or did you feel any tension during the last month?”*), lower in young vs. elderly participants (2.3 ± 1 vs. 2.7 ± 1, respectively, *p* = 0.04; unpaired *t*-test).

#### 3.2.2. Perceived Stress Scale

***Overall population:*** Values of the 10 items at baseline in the overall population are reported in Table 2, together with the total PSS score, which falls in the moderate range of distress (percent of participants showing low, moderate or high levels of total PSS score was 18, 65, and 17%, respectively).

***Sex-related differences:*** Baseline total PSS levels revealed moderate stress in both female and male HCPs (20 ± 7 vs. 21 ± 9, respectively, *p* = ns; unpaired *t*-test; Figure 2). However, the difference between the percent of participants showing high levels of total PSS differ by gender (14 in the female vs. 40% vs. in the male group; χ^2^ test).

Moreover, when compared to males, females showed significantly higher values for item 5 (“*In the last month, how often have you felt that things were going your way?*”; 1.7 ± 0.9 vs. 2.2 ± 0.9, *p* = 0.05; unpaired *t*-test).

***Aging effects:*** The total PSS scores in the two age subgroups fall in the range of moderate stress (21 ± 6 vs. 19 ± 7, young vs. elderly subjects, *p* = ns; unpaired *t*-test; Figure 2). Differences were observed regarding item 4 (“*In the last month, how often have you felt confident about your ability to handle your personal problems?”*) and 7 (“*In the last month, how often have you been able to control irritations in your life?*”), that resulted in higher scores in young vs. elderly participants (1.7 ± 1 vs. 1.37 ± 0.9, *p* = 0.03 and 2.1 ± 0.8 vs. 1.8 ± 0.9, *p* = 0.06, respectively; unpaired *t*-test).

#### 3.2.3. Maslach Burnout Inventory

***Overall population:*** Values of the 22 items, 3 dimensions, and the total MBI score at baseline in the overall population are reported in Table 3 (percent of participants showing low, moderate or high levels were -EE: 47, 19, and 34%; -DP: 57, 25, and 18%; -PA: 46, 23, and 31%, respectively).

***Sex-related differences:*** EE revealed moderate levels in both female and male HCPs (20 ± 12 vs. 22 ± 15, respectively, *p* = ns), as well as DP (5.2 ± 5.7 vs. 8 ± 8, respectively, *p* = ns) and PA (35 ± 8 vs. 37 ± 8, *p* = ns; unpaired *t*-test, Figure 2).

When compared to males, females showed lower values for item 15 (“*I don’t really care what happens to some patients*” in the DP dimension; 0.5 ± 1 vs. 1.2 ± 1.8, *p* = 0.01; unpaired *t*-test).

***Aging effects:*** EE results were moderate in young and older subjects (20 ± 13 vs. 21 ± 12, respectively, ns), as well as PA (34 ± 7 vs. 36 ± 8, respectively, ns), although young participants showed higher levels of DP compared to older HCPs (7.2 ± 6.7-moderate vs. 3.9 ± 5-low, respectively, *p* = 0.002; unpaired *t*-test, Figure 2).

Accordingly, although no difference between the percent of participants showing low, moderate or high levels of EE and PA score were observed, the percent of participants with moderate or high DP differed between the two age groups (58 vs. 27% in young vs. older subjects, *p* = 0.0006; χ^2^ test).

Higher levels were observed in young subjects compared to older ones for item 5 (“*I feel like I treat some patients as if they were objects*”; 1.4 ± 1.8 vs. 0.7 ± 1.2, respectively, *p* = 0.011), 11 (“*I’m afraid this job might harden me emotionally*”; 2.6 ± 2.2 vs. 1.4 ± 1.9, respectively, *p* = 0.002), 15 (“*I don’t really care what happens to some patients*”; 0.7 ± 1.3 vs. 0.3 ± 0.8, respectively, *p* = 0.047), and 22 (“*I feel like patients blame me for their problems*”; 1.1 ± 1.6 vs. 0.4 ± 0.8, respectively, *p* = 0.003), in the “DP” dimension.

Instead, item 7 (*“I effectively address patient problems”*) in the PA dimension results were lower in young participants than in older ones (4.7 ± 1.1 vs. 5.2 ± 1.2, respectively, *p* = 0.007) (unpaired *t*-test).

#### 3.2.4. Reflections

At the end of the burnout questionnaire, there was the option to add a free comment on the perception of the main stressors in response to the following question: “what do you report as your most recent major stressors?”. Although this is not a quantitative and systematic measure, we believe it is interesting to report and comment on this information, which allows for an interpretation of the feelings, frequencies, and intensity with which HCPs described their main perceived stressors. Three main areas were identified, including main stressors related to (a) work, (b) personal, and (c) general stressors (Table 4).

Work-related stressors included system level and relational stressors. System-level stressors are inefficiencies in the work environment that make work more difficult. These factors were beyond HCP control and included burdens of responsibility, high workloads, complex tasks, lack of resources, and time-related stressors. Interactions between team members and relationships with patients (or patient’s families) were the most predominant work stressors reported. Three main team member relationships were highlighted: (a) peer relationship, (b) relationships between higher and lower levels (e.g., supervisor to employer), and (c) relationships between lower and higher levels (e.g., employer to supervisor).

HCP females often reported conflictual, poor, deficient, or incomplete communication. Interestingly, many participants independently proposed corrective actions, suggesting a positive attitude toward problem-solving (e.g., more time to schedule more meetings to resolve communication issues, as well as more clarification, dialogue, and listening to alleviate conflicts with colleagues and better organize activities). Patient-related issues included challenging patients, conflict with patient families, patient deaths, and fear of making mistakes, especially when caring for seriously ill patients or children. Other patient-related concerns included challenging work assignments and the inability to complete the work or not completing it as well as possible.

Individual stressors were self-focused and included emotions related to work, job satisfaction, professional growth, and work–life balance. Job dissatisfaction induced feelings of unappreciation, burnout, inadequacy, low energy, and fatigue, as well as lack of motivation and pleasure, with a sense of loss of empathy towards patients and colleagues. Family difficulties and lack of time for themselves were reported. The lack of time to meet work and home demands was a significant stressor in the women’s personal lives.

Only one HCP reported disappointment with the financial treatment. Moreover, none mentioned other general stressors, such as time spent on business travel or the need to prioritize self-care over work.

### 3.3. MBSR Effects

#### 3.3.1. Psychological General Well-Being Index

**Figure 3:**
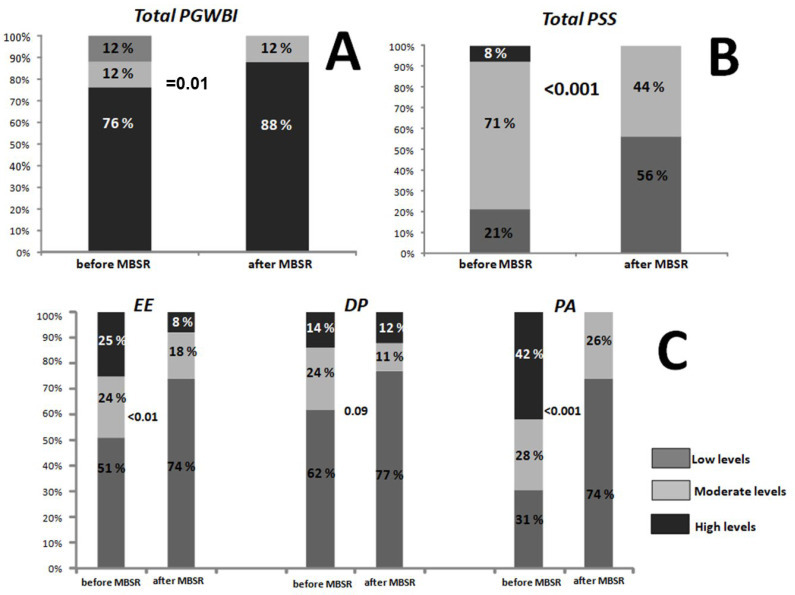
Percentage of participants exhibiting low, moderate or high levels of total PGWBI (**A**), total PSS (**B**), and EE, DP, and PA (**C**) before and after the MBSR program (χ^2^ test). PGWBI: Psychological General Well-Being Index; PSS: Perceived Stress Scale; EE: emotional exhaustion; DP: depersonalization and detachment from the job; PA: lack of personal or professional accomplishment; MBSR: Mindfulness-Based Stress Reduction.

In the subgroup who followed the MBSR training, the total PGWBI score significantly increased from the baseline (81.6 ± 12 and 73.3 ± 14.2, respectively, *p* < 0.001, paired *t*-test). Moreover, the percent of participants exhibiting high levels of PGWBI increased after the MBSR course when compared to the baseline, as reported in Figure 3 (panel A; χ^2^ test).

#### 3.3.2. Perceived Stress Scale

After MBSR intervention, total PSS scores significantly decreased (13.1 ± 5.4 and 18.4 ± 6.5 vs. post vs. baseline, respectively, *p* < 0.001; paired *t*-test). Accordingly, the percent of participants exhibiting moderate or high levels of total PSS decreased after the MBSR course, as shown in Figure 3 (panel B; χ^2^ test).

#### 3.3.3. Maslach Burnout Inventory

After the MBSR intervention, values of EE significantly decreased (12.9 ± 8.6 vs. 18 ± 10.3 at baseline, *p* < 0.001), as well as DP (3.7 ± 4.5 vs. 4.9 ± 5.6 post MBSR vs. baseline, respectively, *p* = 0.03). However, the difference for PA did not reach statistical significance (34.9 ± 7 vs. 36.4 ± 6.7 post MBSR vs. baseline, respectively, *p* = 0.08) (paired *t*-test).

The percent of participants showing moderate or high levels, according to the three principal MBI domain categories, decreased after the MBSR course in any of the groups (Figure 3, panel C; χ^2^ test).

#### 3.3.4. Cardiometabolic Biomarkers

In the subgroup subjected to blood sampling, a significant variation in lipid profile parameters was observed, with significant variations in HDL (63 ± 13 vs. 69 ± 14 mg/dL, *p* = 0.048), LDL (129 ± 29 vs. 110 ± 31 mg/dL, *p* = 0.004), and TotCH/HDL (3.4 ± 0.7 vs. 3 ± 0.5, *p* = 0.006) but not in TotCH (210 ± 34 vs. 200 ± 31 mg/dL) and TG (88 ± 33 vs. 87 ± 27 mg/dL) after MBSR intervention (paired *t*-test).

Levels of hsCRP after MBSR did not vary from baseline (0.34 ± 0.6 vs. 0.43 ± 0.7 mg/dL), as well as glycemia (from 87 ± 6 vs. 89 ± 8 mg/dL) (paired *t*-test).

## 4. Discussion

### 4.1. Baseline Characteristics of the Studied Population

The majority of participants who voluntarily chose to practice MBSR in our study were women. Previous findings have shown that men often hesitate to undertake psychological interventions or to consider emotional adjustment essential to their life satisfaction, while women are actually more inclined to adopt mind–body techniques [26,27]. Mindfulness training and practice can increase adaptation and better control over stress. Thus, it is possible that females are more likely to seek support in situations of distress, which makes them more sensitive and willing to manifest their feelings and thoughts, as previously reported [28]. Moreover, women may have greater interest, understanding, and confidence in the intervention than men, and therefore not only agreed to participate in the program in greater numbers but also engaged more in the program once they decided to participate.

Nonetheless, other important aspects deserve to be further explored in future studies in this context. More precisely, at present very little is known about gender differences in the types of practices, reasons for practicing, or perceived benefits, which are critical to understand how females and males engage in, respond to, and benefit from MBSR intervention. Moreover, more than 50% of the participants were nurses, a profession subjected to shift work and closer contact with patients. Nurses show higher distress scores than physicians or other healthcare professionals in previous studies, and as such, may be more prone to embrace intervention programs that can alleviate stress [29,30,31].

A recent study evidenced how the integration of the Mediterranean diet with mindfulness (and principles related to mindful eating) may reinforce resilience and wellness in adults. In fact, although mindful eating and the Mediterranean diet are not a specific weight-loss program, they may represent general eating behaviors that contribute to a healthy lifestyle by improving psychological status, helping to achieve and maintain an optimal weight. Thus, although this point could not be assessed in our study since all participants followed the principles of the Mediterranean diet [32], the effect derived from the integration of diet and wellness represents an interesting aspect to be explored in future studies on HCPs.

In our population, we found no significant association between physical activity levels and wellness and distress (total PGWBI, total, PSS, MBI-EE, MBI-DP, and MBI-PA; unshown data) at baseline. Interestingly, a recent review examined interventions that combine physical activity and mindfulness training, resulting in effective improvement of mental health and well-being [33]. However, physical activity interventions varied significantly across studies in terms of type, duration, frequency, and intensity [33]. Thus, also in this case, more knowledge is needed to verify the effectiveness of combined approaches (MBSR and physical activity) or a single intervention on distress, as well as to highlight the mechanisms through which they produce beneficial effects on the well-being of HCPs.

Moreover, beyond physical activity, MBSR training could be explored in terms of beneficial changes in health-related behaviors. Surely diet and physical activity, along with smoking and sleep quality are usually the principal aspects contributing to a number of disorders including obesity, metabolic syndrome, type 2 diabetes (T2D), and cardiovascular disease [34,35,36].

### 4.2. Well-Being, Stress, and Burnout

Although our HCPs showed moderate distress levels on average, a percentage of them in our population reported high levels of stress (about 20%, more for EE and PA), which highlights the need to be proactive about this adverse factor and to find strategies to prevent its onset and growth. Nonetheless, other data indicate that the percentage of HCPs being above threshold levels of stress is constantly found around 28%, much higher than that found in the general working population (estimated about 18%), which is similar to the level found in our HCP population [37].

HCP women may show significantly more distress and burnout than their male colleagues. In fact, female healthcare workers often must work more, adding to job demands also family responsibilities such as child or elderly care, housework and other duties that often fall to women [38]. In our study, we observed that the work–family burden has a key influence on the health and well-being of women, as effectively reflected by thoughts reported by our female HCPs, who report work–personal life interference much more frequently than male colleagues. This fact reflects a social “gender asymmetry”, not yet overcome in today’s society with strong repercussions on work and workload. In this context, for several years a company nursery has been operating in our research area, allowing employees to leave their children in a safe environment close to the workplace, reducing concerns about time management and childcare.

Moreover, distress related to relationships and time disposal was strongly perceived by women, although some items relative to PGWBI and MBI are better in female HCPs than in males. It has been suggested that women retain a greater capacity for emotional regulation and cognitive control than men, which may help to reduce undesirable emotional responses and benefit their overall well-being [39,40]. Instead, men are more likely to internalize their stress and articulate their emotions, without consciously perceiving psychological distress as a factor influencing their overall well-being [39,40].

Both female and male reported the burden of high job demands and responsibility, worries of ineffectiveness of care services for the patient, as well as insufficient possibility of professional growth and rewards. Females seem to report higher quantitative and emotional demands in the relationship with colleagues and patients. In particular, one main source of discomfort at work reported by our HCPs was related to relationships with colleagues (with peers and superiors). The reason for this feeling may be related to the lack of autonomy and hierarchical decision-making in many health care organizations. In this context, interventions focused on improving job resources (e.g., social support, autonomy encouragement, feedback on work efficiency and opportunities for work growth) can alleviate the burden of workload on psychological distress.

Young HCPs demonstrated higher levels of stress, likely due to their relatively limited work experience, which can generate greater anxiety and discomfort. As people age, they can progressively readapt their strategies for managing feelings and emotions; thus, older adults can more effectively manage relationships with themselves and others. Reducing the risk of psychological distress for younger HCPs may require change in their organizations, with support and coaching of older workers to develop their job autonomy, encouraging the development of their resources.

### 4.3. MBSR Effects

The present study examined the effects of MBSR intervention on well-being in HCPs both from health biomarkers and psychological points of view, showing positive effects of this practice on well-being, distress, and lipid parameters. Knowledge regarding the complex relationship between mind and body will continue to advance in the coming years. Some previous results analyzed in different meta-analyses provided evidence that mindfulness improves emotional regulation and psychosocial aspects in the general and working populations and in different categories of patients [41,42,43,44].

In particular, some recent data showed that MBSR and other meditation practices provide benefits for stress reduction and well-being in HCPs in different clinical environments, especially in extreme conditions that pose unprecedented challenges on HCPs, such as the COVID-19 pandemic [19,43,44,45]. A systematic review evaluating MBSR effects in intensive care unit nurses (exposed to high stress) evidenced a reduction of emotional exhaustion, depersonalization, and stress-related symptoms [43]. A randomized controlled trial focused on nurses caring for institutionalized elderly people with dementia (*n* = 39 experimental group, *n* = 35 controls) found MBSR effective in reducing compassion fatigue and burnout [44]. Moreover, we and others previously showed the efficacy of MBSR on well-being, burnout, and distress as well as a significant reduction of depressive, anxiety, and somatic symptoms in frontline HCPs during the COVID-19 pandemic [19,45].

Accordingly, we observed a significant improvement in health status through changes in all domains representative of health and psychological status in the health care workers from pre to post MBSR. In this context, structured mindfulness programs appear effective and might be regularly introduced in the routinary work activity in the permanent staff as well as in new hires to favor overall well-being, facing distress and reinforcing concentration and resilience [22].

However, how MBSR programs could be integrated into real occupational health initiatives for healthcare workers remains a challenge, as there are barriers to implementation: these initiatives require support from hospital management, staff time (always in short supply) to fully implement the program, quiet areas suitable for concentration and relaxation, and reinforcement classes or continuous practice (because benefits may decrease with time elapsed) [43].

MBSR practice may also be beneficial to reduce the cardiometabolic risk. Breathing exercises related to MBSR practice may cause changes in the activity of the autonomic nervous system (e.g., increase in vagal tone and reduction in the adrenergic action) [4]. Diaphragmatic breathing practice may reduce stress and cortisol levels [46]. Moreover, physical exercises (e.g., stretching, posture, and flexibility exercises) and improved relationship with food (mindful eating) associated with meditation in the MBSR programs may have beneficial effects on metabolic risk factors, including blood lipid levels [19]. Other available results support the relationship between MBSR and its effects on the autonomic nervous system and benefits on lipids.

A meta-analysis (nine studies) assessing heart rate variability (HRV), which is an established method to evaluate cardiac sympatho-vagal balance, demonstrated that participation in MBSR training improved HRV and the autonomic balance [47]. Interestingly, dysregulation of the autonomic nervous system is associated with the metabolic syndrome and its components; in particular, autonomic nervous system measures (high heart rate and low cardiac autonomic balance) were found to be significant predictors of a 2-year decline in HDL cholesterol in a large general population (1933 participants aged 18–65 years) [48]. Hypothalamic control of hepatic lipid metabolism via the autonomic nervous system is otherwise well known, as sympathetic activity stimulates very low-density lipoprotein (VLDL)-TG secretion, thereby mobilizing TG, whereas parasympathetic activity inhibits VLDL-TG secretion, decreasing TG levels and improving the lipid profile [49].

Our study was not specifically set up to study diabetic patients, in fact none of the subjects enrolled was diabetic and their glucose levels were within the normal reference range; therefore, this fact may be the reason why we did not observe any significant change in blood glucose after MBSR. Thus, although the current results do not exclude a significant effect on T2D subjects, previous data from a meta-analysis suggest that mindfulness programs have the potential to improve glycemic control (as evaluated by hemoglobin A1c-HbA1c) in patients with T2D [50,51].

Regarding CRP, we did not observe any significant change after MBSR. Generally, in previous studies of MBSR intervention, high levels of CRP represented an inclusion criterion, thus a significant effect is possible in such cohorts to which our data cannot be generalized [52]. Interestingly, recent data showed that MBSR reduced cellular proinflammatory gene regulation in older adults, potentially reducing disease risk with key implications for health span, although no effects were observed for circulating protein markers of inflammation (including CRP) [53].

### 4.4. Strengths and Limitations

Strengths of this study were the use of validated psychometric scales, measure of biomarkers, and the implementation of a well-established MBSR program driven by an instructor with high experience. Moreover, we tried to fill some gaps concerning distress according to gender-related differences as well as the paucity of data in older workers with respect to younger people, both in general settings and in HCPs in particular. More importantly, we aimed to evaluate combined psychological and metabolic assessments in HCPs, in order to provide a possible additive cheap tool for a more effective and holistic preservation/achievement of psycho-physical wellness in high distress work environments.

However, there are some limitations which must be taken into account and include the lack of randomization and control as well as the short duration of follow-up. Moreover, although the sample studied may be representative of the hospital staff, it is not large or complete. Thus, it is essential to further confirm present data in larger populations and other settings.

A limited generalizability is due to the predominantly female sample, although it reflects the gender gap in worker composition (majority of women employers) and probably the major disposition of women to follow MBSR training. In particular, females and males as well as subjects belonging to different age categories may have different needs and respond differently to stress, likely requiring the development of tailored programs to best account for these differences. Thus, better knowledge of the relationship between age, gender, and distress can help to plan preventive interventions toward HCPs.

Moreover, although MBSR may be helpful, it is crucial that underlying causes be addressed by activating structural changes, first of all, by enhancing communication between organizational members and a sense of teamwork and leadership (e.g., through periodical team meetings).

Self-selection bias among participants may also exist; participants choose to follow courses, perhaps having particular interest in meditation training. It is therefore possible that the effects of meditation training are different in individuals who are more motivated (placebo-effect-like confounds driven by self-selection), and as such not completely generalizable to programs not requiring self-selected participation.

Other lifestyle variables (e.g., smoking habit and sleep quality) are not considered in the present study but would be incorporated in the future analysis because they may affect the results. Moreover, it would be recommended to increase the sample in number and professional profile, to allow a stratification according to their job burden (e.g., shifts, night work). Other biomarkers which are implicated in the pathogenesis of many chronic conditions (e.g., related to inflammation, oxidative stress) may be also further evaluated.

## 5. Conclusions

In conclusion, the practical implications suggested by the main findings of this manuscript support the integration of meditation into a holistic approach for HCPs. Indeed, the implementation of MBSR programs can improve well-being, by reducing the perception of stress and metabolic disorders, including abnormal levels of lipids, in HCPs. These effects potentially qualify MBSR as a novel complementary strategy for well-being maintenance and cardiometabolic disease prevention.

## Figures and Tables

**Table 1:** Psychological General Well-Being Index results in the overall population.

Items	Mean (SD)
***Anxiety***	
5	3.5 (1)
8	2.5 (1)
17	3.3 (1.1)
19	2.7 (1)
22	2.9 (1.2)
Total score	15 (5)
***Depressed mood***	
3	4.1 (1)
7	3.4 (0.9)
11	4.5 (1)
Total score	12 (2.5)
***Positive well-being***	
1	2.5 (1)
9	2.6 (1.1)
15	3 (1.2)
20	2.7 (1)
Total score	11 (3.5)
***Self-control***	
4	3 (1.2)
14	4.1 (1.4)
18	2.8 (1.1)
Total score	10 (3)
***General health***	
2	3.1 (1.3)
10	3.6 (1.1)
13	3.4 (1.1)
Total score	10 (2)
***Vitality***	
6	3.1 (0.9)
12	2.4 (1.1)
16	2.9 (1)
21	3.3 (0.9)
Total score	11.7 (3.2)
**Total PGWBI score**	70 (16)

Scores of questions 1, 4, 6, 7, 9, 10, 14, 16, 19, and 21 were reversed. PGWBI = Psychological General Well-Being Index.

**Table 2:** Perceived Stress Scale results in the overall population.

Items and Total Score	Mean (SD)
l. In the last month, how often have you been upset because of something that happened unexpectedly?	1.7 (1.2)
2. In the last month, how often have you felt that you were unable to control the important things in your life?	1.9 (1.2)
3. In the last month, how often have you felt nervous and stressed?	2.9 (0.9)
4. In the last month, how often have you felt confident about your ability to handle your personal problems?	1.5 (1)
5. In the last month, how often have you felt that things were going your way?	1.8 (0.9)
6. In the last month, how often have you found that you could not cope with all the things that you had to do?	2.4 (1)
7. In the last month, how often have you been able to control irritations in your life?	1.9 (0.8)
8. In the last month, how often have you felt that you were on top of things?	1.7 (0.8)
9. In the last month, how often have you been angered because of things that happened that were outside of your control?	2.3 (1)
10. In the last month, how often have you felt difficulties were piling up so high that you could not overcome them?	1.8 (1.2)
***Total PSS***	20 (7)

Scores of questions 4, 5, 7, and 8 were reversed.

**Table 3:** Maslach Burnout Inventory results in the overall population.

Items	Mean (SD)
***Emotional exhaustion (EE)***	
1	2.5 (1.8)
2	3.4 (1.5)
3	3 (1.7)
6	1.8 (1.8)
8	2.3 (1.9)
13	1.9 (1.8)
14	2.4 (1.9)
16	1.5 (1.6)
20	1.5 (1.8)
Total score	20 (12)
***Depersonalization and detachment from the job (DP)***	
5	1 (1.5)
10	1.4 (1.8)
11	2 (2.1)
15	0.5 (1.1)
22	0.7 (1.3)
Total score	5.6 (6)
***Lack of personal or professional accomplishment (PA)***	
4	4.6 (1.6)
7	5 (1.1)
9	4.4 (1.6)
12	3.7 (1.5)
17	4.5 (1.4)
18	4.5 (1.5)
19	4.2 (1.6)
21	3.9 (1.8)
Total score	34.7 (7.8)

**Table 4:** Examples of main perceived recent significant stressors as described by HCPs.

Major Areas	Stressor Categories	Specific Stressors	Subjects	Examples of Reported Discomfort
**Work stressors**	Systems-Level Stressors	Work demands and responsibility	both sexes, elderly	#52, male, 67 yrs “I feel discomfort with finding research funding and project deadlines” #94, male, 57 yrs; #123, female, 56 yrs “I feel a burden of responsibility, especially towards my employees”
		Systems-level work barriers	both sexes, elderly	#147, male 46 yrs “increased complexity of work while my ability to respond decreased” #62, female, 53 yrs “fear of lack of tools (time, lack of supplies) that do not allow the patient’s needs to be met” #149, female 58 yrs “excessive workload together with lack of time for myself, misunderstandings between colleagues”
		Time-related stressors	both sexes of various ages	#103, female, 56 yrs “it stresses me out to work quickly and not dedicate the left amount of time to the patient and to preparing the work” #96, female, 50 yrs “time to update superiors and colleagues, to discuss mistakes or positive aspects and to better organize activities”
	Relationships	Team member relationships	females of various ages	#53, female, 53 yrs “stress due to inadequacy of superiors which affects the work process and consequently the quality of care” #131, female, 54 yrs “reduced process optimization—lack of dialogue” #126, female, 42 yrs “hectic work, lack of comparison” #144, female, 37 yrs “relationship with superiors, there is no dialogue” #118, female 52 yrs “I feel little trust from superiors and colleagues with whom there is little communication” #119, female 49 yrs “listening to constant comments from colleagues—interruptions at work—solving problems that I don’t think are within my competence” #146, female, 40 yrs “relationships with superiors, which show they don’t believe in my possibilities” #116, female, 24 yrs “the relationship with the other departments is difficult, they don’t seem to give the left value to my work and don’t try to solve the problems” #87, female, 37 yrs; #92, female, 31 yrs; #143, female, 42 yrs “relationship with patients and colleagues” #74, female, 29 yrs; #75, female 39 yrs; #112, female, 47 yrs “relationships with colleagues”
		Relationships with patients	females of various ages	#145, female, 35 yrs “Patient hostility and lack of appreciation” #120, female, 35 yrs “-being in contact with patients’ pain -not so much the workload but the relationships with colleagues” #100, female, 35 yrs “inadequacy in the face of clinical and human situations, especially when children are involved” #107, female, 40 yrs, “the relationship with the patients stresses me out and I feel exhausted, frustrated and angry at the end of the shift” #68, female, 53 yrs “accept that I can’t do anything, especially for the children. deal with the aggression of parents who accuse you of having made a mistake and blame you for their child’s illness” #69, female, 26 yrs “working with children, relating to the patient’s family, accepting that we cannot do more #115, female, 34 yrs “working in pediatrics means adding the parents’ anxieties and fears to the patient’s well-being. It is not easy to detach oneself from the situations experienced eight hours a day”
**Individual work stressors**	Personal concerns		both sexes of various ages	#122, female, 53 yrs “fear of losing empathy” #140, female 34 yrs “I can’t detach myself from situations with patients and colleagues -I’m detached towards patients -I don’t feel valued -I suffer because my difficulties can weigh on my colleagues” #58, male, 28 yrs “physical tiredness, colleagues’ judgement” #148, female, 35 yrs “relationship with patients: I can’t manage them with the necessary calm and pressure and I don’t feel valued professionally, I’ve lost enthusiasm” #60, female, 25 yrs “fear of making mistakes, lack of confidence in my abilities” #63, female, 56 yrs “sense of inadequacy, relationship with colleagues, stress in seeking control” #67, female, 65 yrs “tiredness, heavy shifts, relatives needing care, children far away, I feel off”
	Professional growth and rewards		both sexes of various ages	#73, male, 55 yrs “grow culturally and be adequate and helpful to patients” #106, female, 32 yrs “I feel like I’m not being rewarded” #139, female, 34 yrs “I love my job, but it stresses me out to have to accept decisions from above without considering the ideas of those who live in the department. I don’t feel valued and I have difficulties in my family where I bring stress and tears”
	Work and family balance		females of various ages	#70, female, 36 yrs “balancing home and work, and not having time for myself” #108, female, 27 yrs; #95, female 49 yrs “reconciling work and family”
**General stressors**	Finances and money		one young male	#86, male 33 yrs “stress from shifts, dissatisfaction with the contractual aspect of the category”

HCPs = health care professionals.

## Data Availability

The original contributions presented in the study are included in the article, further inquiries can be directed to the corresponding authors.

## References

[1] Rink L.C., Oyesanya T.O., Adair K.C., Humphreys J.C., Silva S.G., Sexton J.B. (2023). Stressors Among Healthcare Workers: A Summative Content Analysis. Glob. Qual. Nurs. Res..

[2] Vaccarino V., Bremner J.D. (2024). Stress and cardiovascular disease: An update. Nat. Rev. Cardiol..

[3] Kabat-Zinn J. (2003). Mindfulness-based interventions in context: Past, present, and future. Clin. Psychol. Sci. Pract..

[4] Antonelli M., Donelli D., Gurgoglione F.L., Lazzeroni D., Halasz G., Niccoli G. (2024). Effects of Static Meditation Practice on Blood Lipid Levels: A Systematic Review and Meta-Analysis. Healthcare.

[5] Meyer J.D., Hayney M.S., Coe C.L., Ninos C.L., Barrett B.P. (2019). Differential Reduction of IP-10 and C-Reactive Protein via Aerobic Exercise or Mindfulness-Based Stress-Reduction Training in a Large Randomized Controlled Trial. J. Sport. Exerc. Psychol..

[6] Tang Y.Y., Patterson J.S., Tang R., Chi J., Ho N.B.P., Sears D.D., Gu H. (2025). Metabolomic profiles impacted by brief mindfulness intervention with contributions to improved health. Sci. Rep..

[7] Chen W., Zeng J., Wang W., Yang B., Zhong L., Zhou J. (2020). Comprehensive metabolomic and lipidomic analysis reveals metabolic changes after mindfulness training. Mindfulness.

[8] Ramachandran H.J., Bin Mahmud M.S., Rajendran P., Jiang Y., Cheng L., Wang W. (2023). Effectiveness of mindfulness-based interventions on psychological well-being, burnout and post-traumatic stress disorder among nurses: A systematic review and meta-analysis. J. Clin. Nurs..

[9] Alkhawaldeh J.M., Khawaldeh M.A., Mrayyan M.T., Yehia D., Shudifat R.M., Anshasi H.A., Al-Shdayfat N.M., Alzoubi M.M., Aqel A. (2024). The efficacy of mindfulness-based programs in reducing anxiety among nurses in hospital settings: A systematic review. Worldviews Evid. Based Nurs..

[10] Kajee N., Montero-Marin J., Saunders K.E.A., Myall K., Harriss E., Kuyken W. (2024). Mindfulness training in healthcare professions: A scoping review of systematic reviews. Med. Educ..

[11] Vergeer I., Bennie J.A., Charity M.J., van Uffelen J.G.Z., Harvey J.T., Biddle S.J.H., Eime R.M. (2018). Participant characteristics of users of holistic movement practices in Australia. Complement. Ther. Clin. Pract..

[12] Brown M.M., Arigo D., Wolever R.Q., Smoski M.J., Hall M.H., Brantley J.G., Greeson J.M. (2021). Do gender, anxiety, or sleep quality predict mindfulness-based stress reduction outcomes?. J. Health Psychol..

[13] Upchurch D.M., Johnson P.J. (2019). Gender Differences in Prevalence, Patterns, Purposes, and Perceived Benefits of Meditation Practices in the United States. J. Womens Health.

[14] Rojiani R., Santoyo J.F., Rahrig H., Roth H.D., Britton W.B. (2017). Women Benefit More Than Men in Response to College-based Meditation Training. Front. Psychol..

[15] Tadros M., Newby J.M., Li S., Werner-Seidler A. (2025). A systematic review and meta-analysis of psychological treatments to improve sleep quality in university students. PLoS ONE.

[16] LaMontagne L.G., Doty J.L., Diehl D.C., Nesbit T.S., Gage N.A., Kumbkarni N., Leon S.P. (2024). Acceptability, usage, and efficacy of mindfulness apps for college student mental health: A systematic review and meta-analysis of RCTs. J. Affect. Disord..

[17] Felsted K.F. (2020). Mindfulness, Stress, and Aging. Clin. Geriatr. Med..

[18] Kabat-Zinn J. (1982). An outpatient program in behavioral medicine for chronic pain patients based on the practice of mindfulness meditation: Theoretical considerations and preliminary results. Gen. Hosp. Psychiatry.

[19] Marotta M., Gorini F., Parlanti A., Berti S., Vassalle C. (2022). Effect of Mindfulness-Based Stress Reduction on the Well-Being, Burnout and Stress of Italian Healthcare Professionals during the COVID-19 Pandemic. J. Clin. Med..

[20] Craig C.L., Marshall A.J., Sjöström M., Bauman A.E., Booth M.L., Ainsworth B.E., Pratt M., Ekelund U., Yngve A., Sallis J.F. (2003). International Physical Activity Questionnaire: 12-country reliability and validity. Med. Sci. Sports Exerc..

[21] Sofi F., Macchi C., Abbate R., Gensini G.F., Casini A. (2014). Mediterranean diet and health status: An updated meta-analysis and a proposal for a literature-based adherence score. Public Health Nutr..

[22] Sofi F., Dinu M., Pagliai G., Marcucci R., Casini A. (2017). Validation of a literature-based adherence score to Mediterranean diet: The MEDI-LITE score. Int. J. Food Sci. Nutr..

[23] Seidler Z.E., Wilson M.J., Benakovic R., Mackinnon A., Oliffe J.L., Ogrodniczuk J.S., Kealy D., Owen J., Pirkis J., Mihalopoulos C. (2024). A randomized wait-list controlled trial of Men in Mind: Enhancing mental health practitioners’ self-rated clinical competencies to work with men. Am. Psychol..

[24] Burke A., Lam C.N., Stussman B., Yang H. (2017). Prevalence and patterns of use of mantra, mindfulness and spiritual meditation among adults in the United States. BMC Complement. Altern. Med..

[25] Zimmermann P., Iwanski A. (2014). Emotion regulation from early adolescence to emerging adulthood and middle adulthood: Age differences, gender differences, and emotion-specific developmental variations. Int. J. Behav. Dev..

[26] Dodek P.M., Wong H., Norena M., Ayas N., Reynolds S.C., Keenan S.P., Hamric A., Rodney P., Stewart M., Alden L. (2016). Moral distress in intensive care unit professionals is associated with profession, age, and years of experience. J. Crit. Care.

[27] Johnson-Coyle L., Opgenorth D., Bellows M., Dhaliwal J., Richardson-Carr S., Bagshaw S.M. (2016). Moral distress and burnout among cardiovascular surgery intensive care unit healthcare professionals: A prospective cross-sectional survey. Can. Assoc. Crit. Care Nurses.

[28] Dodek P.M., Norena M., Ayas N., Wong H. (2019). Moral distress is associated with general workplace distress in intensive care unit personnel. J. Crit. Care.

[29] Christodoulou E., Deligiannidou G.-E., Kontogiorgis C., Giaginis C., Koutelidakis A.E. (2024). Fostering Resilience and Wellness: The Synergy of Mindful Eating and the Mediterranean Lifestyle. Appl. Biosci..

[30] Remskar M., Western M.J., Osborne E.L., Maynard O.M., Ainsworth B. (2024). Effects of combining physical activity with mindfulness on mental health and wellbeing: Systematic review of complex interventions. Ment. Health Phys. Act..

[31] Loucks E.B., Schuman-Olivier Z., Britton W.B. (2015). Mindfulness and Cardiovascular Disease Risk: State of the Evidence, Plausible Mechanisms, and Theoretical Framework. Curr. Cardiol. Rep..

[32] Jackson S., Brown J., Norris E., Livingstone-Banks J., Hayes E., Lindson N. (2022). Mindfulness for smoking cessation. Cochrane Database Syst. Rev..

[33] Darehzereshki S., Dehghani F., Enjezab B. (2022). Mindfulness-based stress reduction group training improves sleep quality in postmenopausal women. BMC Psychiatry.

[34] Firth-Cozens J. (2003). Doctors, their wellbeing, and their stress. BMJ.

[35] Xue B., McMunn A. (2021). Gender differences in unpaid care work and psychological distress in the UK Covid-19 lockdown. PLoS ONE.

[36] Goren G., Schwartz D., Friger M., Sergienko R., Monsonego A., Slonim-Nevo V., Greenberg D., Odes S., Sarid O. (2025). Gender Differences in Coping Strategies and Life Satisfaction Following Cognitive-Behavioral and Mindfulness-Based Intervention for Crohn’s Disease: A Randomized Controlled Trial. J. Clin. Med..

[37] Pruessner L., Barnow S., Holt D.V., Joormann J., Schulze K. (2020). A cognitive control framework for understanding emotion regulation flexibility. Emotion.

[38] Verhaeghen P., Aikman S.N., Mirabito G. (2025). Mindfulness Interventions in Older Adults for Mental Health and Well-Being: A Meta-Analysis. J. Gerontol. B Psychol. Sci. Soc. Sci..

[39] Zhang J., Sun J., Zhou Y., Gong L., Huang S. (2025). The effect of mindfulness training on the psychological state of high-level athletes: Meta analysis and system evaluation research. J. Sports Sci..

[40] Zhu M., Wong S.Y., Zhong C.C., Zeng Y., Xie L., Lee E.K., Chung V.C., Sit R.W. (2025). Which type and dosage of mindfulness-based interventions are most effective for chronic pain? A systematic review and network meta-analysis. J. Psychosom. Res..

[41] Chen Q., Liu H., Du S. (2024). Effect of mindfulness-based interventions on people with prehypertension or hypertension: A systematic review and meta-analysis of randomized controlled trials. BMC Cardiovasc. Disord..

[42] Liu M., Guo Y., Bai J., Wang Z., Han J., Zhu J., Wang J. (2025). Effectiveness of mindfulness-based interventions on psychosocial well-being and occupational-related outcomes among nurses in the intensive care unit: A systematic review and meta-analysis. Aust. Crit. Care.

[43] Alharbi B.A.A., McKenna N. (2025). A systematic review of mindfulness-based interventions to reduce ICU nurse burnout: Global evidence and thematic synthesis. BMC Nurs..

[44] Pérez V., Menéndez-Crispín E.J., Sarabia-Cobo C., de Lorena P., Fernández-Rodríguez A., González-Vaca J. (2022). Mindfulness-Based Intervention for the Reduction of Compassion Fatigue and Burnout in Nurse Caregivers of Institutionalized Older Persons with Dementia: A Randomized Controlled Trial. Int. J. Environ. Res. Public Health.

[45] Arts-de Jong M., Geurtsv D.E.M., Spinhoven P., Ruhé H.G., Speckens A.E.M. (2025). Mindfulness-Based Interventions for Mental Health Outcomes in Frontline Healthcare Workers During the COVID-19 Pandemic: A Randomized Controlled Trial. J. Gen. Intern. Med..

[46] Maniaci G., Daino M., Iapichino M., Giammanco A., Taormina C., Bonura G., Sardella Z., Carolla G., Cammareri P., Sberna E. (2024). Neurobiological and Anti-Inflammatory Effects of a Deep Diaphragmatic Breathing Technique Based on Neofunctional Psychotherapy: A Pilot RCT. Stress Health.

[47] Heckenberg R.A., Eddy P., Kent S., Wright B.J. (2018). Do workplace-based mindfulness meditation programs improve physiological indices of stress? A systematic review and meta-analysis. J. Psychosom. Res..

[48] Licht C.M., de Geus E.J., Penninx B.W. (2013). Dysregulation of the autonomic nervous system predicts the development of metabolic syndrome. J. Clin. Endocrinol. Metab..

[49] Bruinstroop E., Fliers E., Kalsbeek A. (2014). Hypothalamic control of hepatic lipid metabolism via the autonomic nervous system. Best Pract. Res. Clin. Endocrinol. Metab..

[50] Hamasaki H. (2023). The Effects of Mindfulness on Glycemic Control in People with Diabetes: An Overview of Systematic Reviews and Meta-Analyses. Medicines.

[51] Ee C.C., Al-Kanini I., Armour M., Piya M.K., McMorrow R., Rao V.S., Naidoo D., Metzendorf M.I., Kroeger C.M., Sabag A. (2025). Mindfulness-based interventions for adults with type 2 diabetes mellitus: A systematic review and meta-analysis. Integr. Med. Res..

[52] Pascoe M.C., Thompson D.R., Jenkins Z.M., Ski C.F. (2017). Mindfulness mediates the physiological markers of stress: Systematic review and meta-analysis. J. Psychiatr. Res..

[53] Lindsay E.K., Marsland A.L., Cole S.W., Dutcher J.M., Greco C.M., Wright A.G.C., Brown K.W., Creswell J.D. (2024). Mindfulness-Based Stress Reduction Reduces Proinflammatory Gene Regulation but Not Systemic Inflammation Among Older Adults: A Randomized Controlled Trial. Psychosom. Med..

